# Demand-related factors influencing caregivers’ awareness of malaria tests and health workers’ testing practices, in Makarfi, Nigeria

**DOI:** 10.1186/s12936-017-2138-8

**Published:** 2017-12-12

**Authors:** Olufemi Ajumobi, Kabir Sabitu, IkeOluwapo Ajayi, Patrick Nguku, Joy Ufere, Peter Wasswa, Chinwoke Isiguzo, Jennifer Anyanti, Jenny Liu

**Affiliations:** 1 National Malaria Elimination Programme, Abuja, Nigeria; 2 Department of Community Medicine, Ahmadu Bello University, Zaria, Nigeria; 3 Department of Epidemiology and Medical Statistics, University of Ibadan, Ibadan, Nigeria; 4 Nigeria Field Epidemiology and Laboratory Training Programme, Abuja, Nigeria; 5 African Field Epidemiology Network (AFENET) - Nigeria Country Office, Abuja, Nigeria; 6 World Health Organization, Abuja, Nigeria; 7 Makerere University School of Public Health, Kampala, Uganda; 8 Society for Family Health, Abuja, Nigeria; 9 University of California, San Francisco (UCSF), San Francisco, CA USA

**Keywords:** Malaria testing, Healthcare workers, Children, Nigeria

## Abstract

**Background:**

Despite the World Health Organization’s recommendation of malaria test-treat strategy, which is the treatment of parasitological confirmed malaria cases with anti-malarials, presumptive diagnosis of malaria remains fairly common in Nigeria. The reasons for this have not been established in Makarfi, Nigeria, despite the high burden of malaria in the area. A study was conducted among caregivers of febrile children less than 5 years presenting for treatment to understand their awareness of malaria diagnostic testing and being offered testing by clinicians, the determinants of these outcomes, and caregivers’ perspectives of health workers’ testing practices.

**Methods:**

Using mixed-methods, data was combined from sub-analysis of cross-sectional survey data (n = 295) and focus group discussions (n = 4) with caregivers conducted in Makarfi General Hospital (Kaduna State, Nigeria) and surrounding communities in 2011. Bivariate and multivariate analysis of the quantitative survey data was conducted to examine associations of caregivers’ sociodemographic characteristics with testing awareness and having ever been offered testing. Transcripts from focus group discussions (FGD) were analysed for emerging themes related to caregivers’ perspectives on malaria testing.

**Results:**

Among surveyed caregivers who were predominantly female (81.7%), not formally educated (72.5%), and were housewives (68.8%); only 5.3% were aware of any diagnostic testing for malaria, and only 4.3% had ever been offered a malaria test by a health worker. Having at least a primary level education (adjusted odds ratio [aOR] 20.3, 95% CI 4.5–92.1) and living within 5 km of the hospital (aOR 4.3, 95% CI 1.5–12.5) were determinants of awareness of malaria testing. Also, these were determinants of previously having been offered a test (aOR 9.9, 95% CI 2.1–48.7; and aOR 4.0, 95% CI 1.1–14.7). FGD showed many caregivers believed that malaria testing was for severe illness only, and that proximity to a health facility and cost of treatment influenced the seeking and receiving of care.

**Conclusions:**

Uptake of malaria testing prior to treatment can be improved by increasing its awareness and addressing misunderstandings among caregivers, promoting testing practices among health workers, and availing caregivers living farther from health centres alternative opportunities for community case management of febrile illnesses.

## Background

In Nigeria, presumptive treatment of febrile illnesses is a common practice at homes and in clinical settings which increases the risk of developing drug resistance for currently used anti-malarial drugs [1]. Globally, adherence to confirmatory laboratory malaria diagnosis in all ages falls short of expectation. In 2015, the percentage of suspected malaria cases that received a malaria diagnostic test was 74 and 76% globally and in the African region, respectively [2].

In 2010, the national guidelines for malaria diagnosis and treatment have been revised to recommend universal testing of all fever cases irrespective of age and administration of artemisinin-based combination therapy to confirmed uncomplicated malaria cases in line with World Health Organization (WHO) guidelines [3–5]. This contrasts with clinical diagnosis of malaria, which is based on non-specific signs and symptoms, often times the presence of fever, without a diagnostic test [4]. Despite the change in national guidelines, changes in practices to adhere to the new guidelines among health workers may not have immediately occurred and malaria testing remains low.

The reasons for this trend is not clear. Notably between 2010 and 2017, the National Malaria Elimination Programme (NMEP) has deployed over 68 million malaria rapid diagnostic tests (RDTs), an easy-to-use test that gives results within 15–20 min, in recent years in efforts to increase confirmatory testing before treatment and early identification of non-malarial febrile illnesses [6–8].

While a number of studies have documented suboptimal care-seeking for malaria diagnosis before treatment in Nigeria, information on the level of awareness for malaria testing among consumers, and health workers’ testing practices, and their determinants is lacking [9, 10]. Understanding these factors may help to identify where interventions should be targeted to change presumptive treatment behaviours and increase the use of diagnostic testing for malaria.

This paper aims to fill this gap by examining the determinants of testing awareness among caregivers of febrile children and whether or not they are offered testing by health workers when seeking malaria treatment. The data were drawn from a larger research project that evaluated the diagnostic performance of RDTs in febrile children under the age of 5 years (U5) in Kaduna State, Nigeria, in 2011 and the associated parasite-specific and seasonal factors that could influence the test performance [11]. For this study, the results of the survey data administered to caregivers presenting for treatment at the local general hospital specifically on testing practices were analysed and supplemented with further insights on caregivers’ perspectives on malaria testing obtained from focus group discussions (FGDs) conducted in the hospital’s surrounding communities. The results can inform the deployment of interventions that will address both caregiver care-seeking behaviour and health workers’ practices towards malaria testing.

## Methods

### Study area

This mixed method sub-study is part of a larger study that assessed diagnostic performance of RDTs in febrile children conducted in Makarfi, Kaduna State, in northwest Nigeria in 2011, which has been partly described elsewhere [11]. Makarfi has a high malaria prevalence of 44% [12]. The population in and around Makarfi is predominantly Muslim and most households are engaged in farming. For women and children, the decision to seek care outside of the home is usually made by the male head of household. The quantitative component of this sub-study, a hospital-based cross-sectional survey of caregivers of febrile children U5 presenting for treatment at Makarfi General Hospital (MGH), included questions about utilization of and reasons for non-use of laboratory services for malaria diagnosis. Additionally, focus group discussions (FGDs) were conducted to further explore underlying factors influencing diagnostic care-seeking. Within the hospital’s catchment area, FGD participants were drawn from two purposefully selected communities, Kuruntumawa (rural, 45 km from MGH) and Makarfi (semi-urban, encompassing MGH).

At the time of the study, Makarfi General Hospital was a 51 bed-hospital with two medical doctors providing malaria case management services. Average patient load was 400 persons per month, of which about 50 were children below 5 years. There were eight laboratory (lab) staff (three laboratory scientists, two laboratory technicians and three assistants). Of these, six had ≥ 5 years of experience and the two had 4–5 years of experience. Two of the staff were trained on malaria microscopy using the strip method as part of the undergraduate curriculum; none had received any formal training on malaria microscopy or use of RDTs. The hospital had a laboratory with two binocular microscopes. Standard operating procedures were implemented, and basic equipment and materials were available. Though the laboratory staff reported average request for malaria microscopy was 3–5 examinations per day and 6–10 slides per day were made, there were no RDTs or basic Giemsa stain for malaria microscopy. Control slides were examined by the principal investigator, but there was no quality assurance mechanism in place for malaria microscopy.

### Sample size determination

A minimum sample size of 175 was calculated based on [13]

$${\text{N}} = \frac{{( {1.96 + 1.28})^{2} \times P( {1 - P} )}}{{{{\left( {P - P{\text{o}}} \right)} /{P{\text{x}}}}}}$$

using a malaria prevalence (*P*x) of 48% [14], estimated malaria RDT sensitivity of 85% (*P*) and minimum sensitivity (*P*o) of 95%. Overall, 300 study participants were recruited [11].

### Quantitative data and analysis

The primary caregiver of each of 300 eligible U5 children with fever (≥ 37.5 °C) or history of fever and consented to participate in the study were consecutively selected and enrolled as they presented for treatment. Five caregivers were excluded because of incomplete and missing data, resulting in 295 completed caregiver surveys (for further details see [11]). The quantitative data for this sub-study was collected using a standardized, pre-tested questionnaire administered to caregivers by a bilingual study interviewer from December 2010 to August 2011. The questionnaires were translated into the local Hausa language, the predominant spoken language, and back-translated into English to avoid any ambiguity. The survey was designed to collect information on clinical and pre-treatment history of patients, caregivers’ awareness of malaria tests and whether or not they were offered and took malaria test for the child’s previous episodes of fever in the past year. Basic sociodemographic and economic background characteristics (i.e., sex, education, occupation, average monthly income earning) were also collected, including access to health facilities within 5 km or a 30-min walk from their residence, to understand the context for care-seeking.

The two main outcomes of interest were coded as follows:

- *Awareness of any malaria testing* reflects participants’ responses to the question, “Have you ever heard about any test for the detection of malaria?” coded 1 for “Yes” and 0 for “No.”
- *Offered a malaria test* reflects responses to the question, “When you brought your child with fever to the heath facility, have you ever been asked by a health worker to do a laboratory test for malaria?” coded 1 for “Yes” and 0 for “No”.

Although, caregivers were also asked whether or not they actually had the test performed when offered, all (n = 11) those who answered “Yes” were the same respondents who answered “Yes” to the question of being “offered a malaria test,” and this outcome was not analysed separately.

Univariate and bivariate analysis of the data were conducted. First, the sociodemographic characteristics and univariate results for caregivers’ awareness of and being offered a malaria test were described. A bivariate analysis was conducted of the associations between sex, education (i.e., none or informal education vs. at least primary education), occupation (i.e., housewife, farmer, civil servant, and others), and distance from nearest health centre (i.e., < 5 km vs. > 5 km) with each of the outcomes. Determinants of the outcomes were ascertained at multivariate analysis.

### Qualitative data and analysis

Four FGD sessions with two independent groups of men and women were conducted in the local language (Hausa) by a team of trained, bilingual research assistants (moderator, note-taker and observer) in the Makarfi and Kuruntumawa communities. Eligible participants, living in Makarfi and Kuruntumawa communities, aged 18–35 years, and who have ever cared for a febrile child U5 were identified by convenience sampling through the local development committees after seeking permission from the community head. Those who were willing to participate were stratified by sex to account for cultural sensitivity. Seven caregivers of each sex who had ever cared for a child with fever below 5 years, were selected in each community to participate in separate male and female FGD sessions, each lasting between 60 and 90 min. Research assistants trained and experienced in conducting FGDs used a semi-structured discussion guide to explore perceptions, beliefs, and practices related to malaria diagnosis and treatment for a child with suspected malaria. The discussions for each session were audio-recorded and the note-taker also wrote down observations and discussion points. Interviewers transcribed the voice recording and translated the notes into English language immediately after each session.

The FGD notes were studied carefully and analysed using detailed content analysis comprised of: familiarization, identification of themes, indexing, charting, mapping and interpretation [15]. Related to malaria testing, key themes identified included awareness and uptake of malaria testing, determinants of malaria testing and of ‘being offered a malaria test’. Results are presented in narratives.

## Results

### Respondent characteristics

In the caregiver survey sample (Table 1), 241 (81.7%) were women, 203 (68.8%) were housewives, 65 (22.0%) were farmers, 10 (3.4%) civil servants, 214 (72.5%) were not educated or had informal education, 81 (27.5%) had at least primary education, and 241 (81.7%) lived within 5 km of MGH. There were only five responses to average monthly income earning. Of these two earned > 5000 ($32), one person each earned < 1000 ($6.4) and 1000–5000 ($6.4–$32) and one earned nothing ($1: 155.71 at the time of the study). Of the 28 FGD participants, 21 (75%) were not formally educated while seven (25%) had at least primary school education. All women (n = 14) who participated in FGD sessions had informal education (Table 2).

**Table 1:** Characteristics of consented caregivers of febrile children presenting at Makarfi General Hospital, Makarfi, Nigeria (N = 295)

	Frequency	Percent
A. Characteristics		
Sex		
Male	54	18.3
Female	241	81.7
Occupation		
Housewife	203	68.8
Farmer	65	22.0
Civil servant	10	3.4
Others^a^	17	5.8
Highest educational attained		
None/informal	214	72.5
Primary	38	12.9
Secondary	35	11.9
Tertiary	8	2.7
Distance from MGH (km)		
< 5	54	18.3
≥ 5	241	81.7
B. Outcomes		
Aware of malaria test (N = 283)		
Yes	15	5.3
No	268	94.7
Ever asked to do the test (N = 259)		
Yes	11	4.3
No	245	95.7

^a^Include: artisans, students, traders

**Table 2:** Characteristics of focus group discussion participants, Makarfi, Nigeria

Community	Type of community	Educational status	Sex (n)	
			Male	Female
Makarfi	Semi-urban	Nil	0	7
		Islamiyya	2	
		Primary school	2	
		Secondary school	3	
Kuruntumawa	Rural	Islamiyya	5	7
		Primary school	1	
		Secondary school	1	

### Awareness and uptake of malaria testing

Of the 283 caregivers who responded to the question “Have you ever heard about any test for the detection of malaria?” only 15 (5.3%) caregivers were aware of malaria testing. Of the 259 who responded to the question “When you brought your child with fever to the heath facility, have you ever been asked by a health worker to do a laboratory test for malaria?” 11 (4.3%) caregivers had ever been offered a malaria test by a health worker (Table 1). All who were offered malaria test reported accepting the test and having the test performed on their child.

### Determinants of malaria testing

Results of bivariate and multivariate analyses of survey data on awareness of malaria testing and being offered a malaria test are presented in Table 3. Awareness of malaria test was significantly associated with having at least primary education [OR 20.7, confidence interval (CI) 4.6–94.3] and living close to hospital [odds ratio (OR) 4.2, CI 1.5–12.2]. The likelihood of being offered a malaria test was associated with higher education of caregivers (OR 13.2, CI 2.8–62.8) and closer proximity to hospital (OR 6.6, CI 1.9–22.8).

**Table 3:** Association between sociodemographic characteristics, awareness and offering of a malaria test to caregivers of febrile children, Makarfi, Nigeria

Characteristic	Awareness of any malaria testing (N = 283)				Offered and did test [previous experience]^a^ (N = 259)			
	Yes	No	OR (95 CI)	aOR	Yes	No	OR (95 CI)	aOR
Sex								
Male	4	47	1.701 (0.522–5.603)	–	1	48	0.417 (0.052–3.334)	–
Female	11	221			10	200		
Occupation								
Housewife^c^	9	187	(0.622 to [− 1.000])^d^	–	9	163	3.313 (0.411–26.707)	–
Farmer^b^	0	61			1	60		
Education								
At least primary	13	64	20.720 (4.555–94.250)	20.265 (4.461–92.059)	9	63	13.210 (2.781–62.790)	9.907 (2.104–48.728)
Nil/informal	2	204			2	185		
Distance (km)								
< 5	7	46	4.223 (1.459–12.220)	4.329 (1.499–12.500)	6	38	6.632 (1.927–22.830)	4.000 (1.095–14.706)
≥ 5	8	222			5	210		

^a^Previous experience means ever asked to do a malaria test

^b^61 responses

^c^196 and 172 responses for awareness and previous experiences respectively

^d^Fisher-Exact

Having at least a primary level education (adjusted OR [aOR] 20.3, 95% CI 4.5–92.1) and living within 5 km of the hospital (aOR 4.3, 95% CI 1.5–12.5) were determinants of awareness of malaria testing. Also, these were determinants of previously having been offered a test (aOR 9.9, 95% CI 2.1–48.7; and aOR 4.0, 95% CI 1.1–14.7).

From the FGDs, factors mentioned by caregivers as influencing utilization of malaria health services were similar to the survey results. The lack of demand for malaria testing was overall linked to poor knowledge of malaria case management among caregivers. The most frequently mentioned factor influencing use of malaria tests was lack of awareness of malaria tests. FGD participants indicated that testing was generally an unknown step in the care-seeking process:

*“We are not aware, we just complain to doctor and medication is given to us, simple!”*

(male, 35 years, farmer, semi-urban).

*“Only few of us knew about blood test to diagnose malaria”*

(female, 25 years, housewife, semi-urban).

Other factors included some misunderstandings about when and under what conditions a malaria test is needed.

*“Blood test is done only when patients present with severe illness and loss of weight”*

(male, 23 years, farmer, rural).

*“It is only performed for blood transfusion”*

(male, 25 years, business owner, semi-urban)

Even when caregivers were offered testing, some reported they were not told about the purpose of tests.

*“Yes, they ask us to do test but we don’t know if it’s for malaria”*

(male, 28 years, farmer, rural)

Bivariate and multivariate analyses generally indicated that caregivers living farther away from the health facility were less likely to be aware of malaria testing (Table 3). FGD participants indicated that self-treatment at home or with medicines purchased from patent medicine stores close to their home was a typical practice prior to seeking care farther away at the facility.

*“Home treatment first, before going to the hospital”* (female, 25 years, housewife, semi-urban).

*“Some people used to go to any chemist shop and buy Panadol* (*Acetaminophen*) *tablets* etc*.”* (male, 30 years, farmer, rural).

### Determinants of being offered a malaria test

Few caregivers reported ever having been offered a malaria test by a health worker (Table 1). FGD participants indicated that malaria tests were not usually requested by health care workers who, instead, often gave treatment based on symptoms. Moreover, health care workers often relied on clinical diagnosis rather than laboratory testing.

*“When we present our complaints they give us drugs and asked us to go”* (female, 35 years housewife, semi-urban).

*“No blood test is done before giving drugs”* (female, 26 years, petty trader, rural).

While caregivers deferred to doctors’ decisions, other factors that influenced malaria testing included cost and non-availability or lack of access to services. In all FGD sessions, undergoing malaria diagnosis was linked to the cost of diagnostic services.

*“If the doctor asked us and if the services are available and affordable, we shall have to do it”* (male, 30 years, farmer, semi-urban).

*“The cost isn’t within the reach of the common man”* (male, 30 years, farmer, semi-urban)

*“It will depend on money, that is, if one has the money to buy drugs and do the test, one will”* (male, 35 years, farmer, semi-urban)

Notably, higher education of caregivers was linked to increased likelihood of being offered a malaria test (OR 13.2, CI 2.8–62.8) (Table 3). Similarly, caregivers living in closer proximity to the more affluent urban health centre were more likely to be offered a test (Table 3). FGD respondents indicated that access and availability of diagnostic services were important limiting factors for undergoing testing.

*“If the services are far from us we won’t be able to use it”* (male, 30 years, farmer, rural).

*“It is only at Makarfi main town that laboratory services are available”*

(female, 18 years, petty trader, rural)

*“The services are not available in the community”* (male, 27 years, farmer, semi-urban)

### Caregivers’ recommendations for addressing identified barriers

FGD respondents overwhelmingly recommended carrying out extensive awareness campaigns in the community on the availability and need for malaria laboratory services at health facilities and training of health workers on parasite-based diagnosis, provision of laboratory and ancillary services at the health centres, and provision of quality drugs and services at affordable cost in rural communities.

“*Create more awareness on the importance of laboratory services”* (18 years, housewife, rural)

*“Government to provide free drugs, more equipment and trained staff, in all the facilities in the local government area (LGA)”* (male, 35 years, semi-urban)

*“Government to make laboratory services available in all the clinics in the town and villages”* (35 years, housewife, semi-urban)

*“Government to make services free for both women and children in all the health facilities [urban and rural]”* (female, 28 years, petty trader, semi-urban)

## Discussion

This study aimed to understand awareness of malaria testing, health workers’ offering of malaria tests, and the associated factors among caregivers of febrile children U5. Overall, relatively few caregivers were aware of malaria testing and health workers’ offering of malaria tests. Lack of malaria testing initiated by health workers is a major contributor to the continued practice of presumptive treatment of febrile cases, and thus a barrier to parasitological diagnosis for malaria. Several studies have shown that health workers under-request malaria tests and overly relying on clinical judgement [16, 17], significant barriers to appropriate utilization of laboratory services at health facilities. Similar studies in Ghana (10%), Kenya (20%) and Tanzania (26%) have reported low health worker malaria testing practices [18–20]. Availability and cost of the malaria test, health workers training, follow-up supportive visits and supervision after initial training, have been identified as major factors influencing malaria testing practices [19, 21].

Presumptive treatment of uncomplicated malaria remains an ingrained behaviour among health workers [22, 23] and caregivers [24] that is difficult to change. Furthermore, because caregivers tend to defer to the expertise of doctors, improving testing practices among health workers should be prioritized while increasing awareness among caregivers can further help to reinforce the importance of testing before treatment. At the time of the study, there were no awareness campaigns, which contributed to low awareness of testing importance or availability. Moreover, since then, there have been efforts to increase awareness campaigns (TV media, community mobilization) in Kaduna state with support from The Global Fund grant and UK Department for International Development-Support to National Malaria Programme project up till mid-2016. Though, currently there is no TV programme that promotes health campaigns on malaria in Kaduna state; moreover, NMEP has a social media page dedicated to engaging the public on malaria interventions and has planned for social behavioural change activities including health campaigns in the new Global Fund grant commencing in 2018.

The percentage of clinically diagnosed malaria cases decreased from 74% (2013), to 37% (2014) and 27% (2015) [25]. This recent declining trend in clinical diagnosis of malaria based on the National Health Management Information System (NHMIS) data though not very clear, may be attributed to capacity building of health workers on malaria case management at all levels and public awareness on malaria testing over the years. The malaria data from 2010–2011, which were a mix of presumptive and confirmatory malaria diagnosis (cannot be distinguished), were not readily available in NHMIS. The 2010–2011 data was a desk-version of NHMIS which is defunct. In 2013, the NHMIS underwent transformation into online District Health Information System platform which excluded historical malaria data including 2010–2011 malaria data.

Having at least primary education was also significantly associated with the likelihood of being aware of a malaria test and being offered a test as well as a determinant of these, which may suggest that healthcare workers may selectively offer testing based on their perception of the caregiver’s ability to pay or comprehend. Education plays a central role in demanding for services and health workers are likely to offer insight into a test if they perceive the patients can comprehend [26]. Commonly the more educated a caregiver is, the higher her socioeconomic status. Healthcare workers often anticipate the ability of the caregiver to pay and probably treat better educated person in higher socioeconomic status differently and thus are more likely to offer their children malaria tests [24]. There is a positive correlation between low education and poverty, and poverty restricts access to health services [27].

Living in close proximity to the hospital was significantly associated with having heard about a malaria test and being offered a malaria test, and also a determinant of these. This further underscored the importance of distance as a driver for caregivers’ seeking care. If the patient is from afar, health workers may not be disposed to offer malaria testing, which adds to cost of transport and might discourage next visit for similar illness. Health workers may treat presumptively as a trade-off between being able to afford medicines and both medicines and malaria testing among patients perceived to be poorer. In this study, unemployed housewives (68.8%) and peasant farmers (22.0%) who are in the low socioeconomic stratum, constitute the majority (91%) of the respondents. The 2013 Nigeria Demographic and Health Survey [28] and other studies revealed that the common impediments to accessing health care in Nigeria included inadequate information, financial barriers, and distance to a health facility [29, 30]. These access barriers can be overcome by ongoing community-based initiatives such as integrated community case management of childhood illnesses. Self-treatment at home or with medicines purchased from patent medicine stores were common probably because of distance from the health facility. A previous survey revealed more than half of the caregivers of febrile children sought advice or treatment from chemist shops/patent medicine vendors [31].

Largely, the malaria programme in Nigeria is donor-driven. Currently, there is no government or donor fund for socio-behavioural change activities and community case management because of new focus of present Global Fund grant extension towards mass long-lasting insecticidal net campaign in 2017, and closure of major donor-funded malaria projects for over a year. There is an urgent need to reinvigorate these initiatives to sustain the gains in malaria control and eventual elimination.

The percentage of suspected malaria cases receiving a malaria test decreased from 64% in 2013, to 51% in 2014, and 47% in 2015 suggesting that overall diagnostic testing by parasitological confirmation is declining [25]. The reason for the declining overall trend in testing reported in NHMIS is not very clear. This may be due to lack of consistent availability of RDTs and health workers’ concerns on diagnostic accuracy of RDTs [7, 11, 19, 21]. This is one of the few mixed-method studies that have documented barriers to malaria testing. Previously, NMEP has embarked on deployment of community volunteers to increase access to testing with support from the Global Fund, but this was not scaled-up sufficiently and is defunct. Caregivers needs to be empowered with information to demand for malaria testing and not defer to health workers’ direction towards clinical diagnosis. Cost of malaria testing remains a deterrent to testing especially when caregivers pay out of pocket in facilities where tests are not affordable. This study is one of the few mixed-method studies that reported misunderstandings about malaria testing. Hitherto, NMEP is not aware and has not developed a strategy to address this. The paper will drive implementation of effective strategies for enhanced uptake of malaria testing in Nigeria and elsewhere.

The limitations of this study should be noted. The investigators were unable to investigate socio-cultural determinants, perspectives of health workers and other supply-side barriers to malaria testing. The influence of perceived cost on use of test was not explored. Generally, expression of income earnings is a very sensitive issue and this might have accounted for few responses from civil servants, a minority of respondents interviewed. Predominantly, the respondents were housewives who do not earn an income and farmers whose income depends on seasonal harvest. The findings of this study might not be generalizable because only issues among patrons of public sector services, which represent a minority of healthcare provision for malaria in Nigeria, were examined [28].

## Conclusions

Few caregivers in semi-urban/rural communities in Makarfi Nigeria, were aware of malaria testing for febrile illnesses among children under 5 years of age. Lack of awareness of malaria testing and being offered the test by health workers were associated with low education status and long distance from health centres in quantitative analysis; qualitative data showed that cost, misunderstandings about when testing is needed, lack of explanation from health workers, and deference to health workers’ directions were also related factors. These results along with caregivers’ own recommendations on how to address barriers to malaria testing suggest that both health workers and caregivers need to be sensitized to regular parasitological testing for malaria as a core component of fever management. Further, community-based initiatives for malaria testing and treatment may be considered as one way to address the costs of care-seeking that FGD respondents cited as impediments.
